# Author Correction: Solution-processed hybrid perovskite photodetectors with high detectivity

**DOI:** 10.1038/s41467-019-09822-6

**Published:** 2019-04-18

**Authors:** Letian Dou, Yang (Micheal) Yang, Jingbi You, Ziruo Hong, Wei-Hsuan Chang, Gang Li, Yang Yang

**Affiliations:** 1 Department of Materials Science and Engineering, University of California,, Los Angeles, California 90095 USA; 2 California NanoSystems Institute, University of California, Los Angeles, California 90095 USA

Correction to: *Nature Communications* 10.1038/ncomms6404, published online 20 November 2014

This Article contains an error in Equation 2 in that the denominator is inverted. The correct form of Equation 2 is: $D^ \ast = \frac{{\left( {{\rm{A}}f} \right)^{1/2}}}{{\left( {i_n{\mathrm{/}}R} \right)}}$. The error has not been fixed in the PDF or HTML versions of the Article.

